# The Puerto Rico community engagement alliance (PR-CEAL) against COVID-19 disparities: outreach and research engagement efforts in disproportionately affected communities

**DOI:** 10.3389/fpubh.2024.1420270

**Published:** 2024-07-18

**Authors:** Adriana D. Pons-Calvo, Cynthia M. Pérez, Karelys Canales-Birriel, Zaydelis Tamarit-Quevedo, Norangelys Solís-Torres, Andrea López-Cepero, Enid García-Rivera, María Larriuz, Edna Acosta-Pérez, Marcilyn Colón, Zuleska Soto Román, Ana P. Ortiz, Fabiola Rivera-Gastón, Vivian Colón-López

**Affiliations:** ^1^Cancer Control and Population Sciences Division, Comprehensive Cancer Center, University of Puerto Rico, San Juan, Puerto Rico; ^2^Department of Biostatistics and Epidemiology, Graduate School of Public Health, University of Puerto Rico, San Juan, Puerto Rico; ^3^Department of Epidemiology, School of Public Health, Emory University, Atlanta, GA, United States; ^4^Endowed Health Services Research Center, School of Medicine, University of Puerto Rico, San Juan, Puerto Rico; ^5^Center for Sociomedical Research and Evaluation, Graduate School of Public Health, University of Puerto Rico, San Juan, Puerto Rico; ^6^The Hispanic Alliance for Clinical & Translational Research in Puerto Rico (Alliance), San Juan, Puerto Rico; ^7^Department of Social Sciences, Graduate School of Public Health, University of Puerto Rico, San Juan, Puerto Rico

**Keywords:** COVID-19, vaccine, disparities, outreach, research, prevention, Puerto Rico

## Abstract

In September 2020, the National Institutes of Health acted in response to the COVID-19 pandemic, recognizing the critical need to combat misinformation, particularly in communities disproportionately affected by the crisis. The Community Engagement Alliance (CEAL) emerged as an initiative dedicated to fostering reliable, science-based information, diversity, and inclusion; aiming to implement effective strategies to mitigate the spread of COVID-19 nationwide. One of the teams participating in this initiative is Puerto Rico-CEAL (PR-CEAL). Our whose goal was to raise awareness about the coronavirus disease and advance research, mainly focusing on vulnerable and underserved populations. This concept paper seeks to outline PR-CEAL’s infrastructure during its initial two cycles, providing insights into the research and community engagement activities designed to enhance prevention, counter misinformation, and foster awareness and uptake of COVID-19 vaccines. Ultimately, our objective is to reflect on the strengths and challenges encountered thus far as we endeavor to sustain this robust infrastructure, addressing ongoing public health issues with a forward-looking approach.

## 1 Introduction

On March 11, 2020, the World Health Organization officially declared COVID-19 a pandemic (1). Since then, global efforts have combated the spread of SARS-CoV-2 relentlessly. In the United States (US), disturbingly, studies consistently reveal striking disparities, with Black and Hispanic individuals facing a two-fold higher risk of COVID-19-related mortality and a four-fold higher likelihood of hospitalization compared to their White counterparts (2–5). In response to these alarming inequities and the pressing need to combat COVID-19 and vaccine misinformation, the National Institutes of Health (NIH) launched the Community Engagement Alliance (CEAL) Consortium. Eleven states received grants on September 16, 2020; by April 2021, 10 additional states and jurisdictions had been funded. Puerto Rico CEAL (PR-CEAL) emerged as part of the second cohort of consortiums awarded (6, 7). This nationwide initiative aimed to raise awareness, foster educational research, and address outreach and engagement in communities disproportionately affected by the pandemic (7–9).

This community case study outlines the infrastructure of PR-CEAL and the research efforts that have laid the foundation for establishing a targeted community engagement and research network. Furthermore, it aims to reflect on the program’s strengths and challenges as it strives to sustain its infrastructure while continuing to address critical public health issues among medically underserved minority groups disproportionately burdened by disease, hospitalizations, and mortality.

## 2 Context

According to the 2020 US Census, Puerto Rico’s population was estimated at 3.2 million people, with a median age of 42.4 years and a median household income of $21,967, significantly lower than the United States’ median household income of $61,521 (10). Preceding the pandemic, the enduring impact of environmental disasters on the island had already taken a toll on its economy, health, and security, triggering a substantial migration of Puerto Ricans to the mainland United States (11–18). In addition, the population in Puerto Rico has dwindled by an estimated 4.4% since 2017 (17). This migration dynamic extended to healthcare professionals, with approximately 15% of medical specialists leaving in pursuit of enhanced resources and salaries in the US (16, 18).

The COVID-19 pandemic further compounded the cumulative effects of socioeconomic issues and environmental disasters over the preceding 6 years in Puerto Rico, exerting a detrimental impact on morbidity and mortality in the island. This sequence of events resulted in what can be described as “parallel pandemics,” exacerbating socioeconomic and health inequalities (19). Despite facing these challenges, Puerto Rico boasted one of the lowest rates of confirmed cases and deaths related to COVID-19 in the US. As of June 30, 2023, Puerto Rico had reported 1,285,787 confirmed cases and 6,099 deaths (20). This success is attributed to an effective collaborative partnership between academic institutions, government entities, and community-based organizations. This collaborative effort has proven instrumental in implementing preventive and educational initiatives, playing a crucial role in mitigating the burden of COVID-19 in underserved communities on the island (3).

## 3 PR-CEAL infrastructure

### 3.1 PR-CEAL Cycle 1: September 2021–March 2022

**Figure 1:**
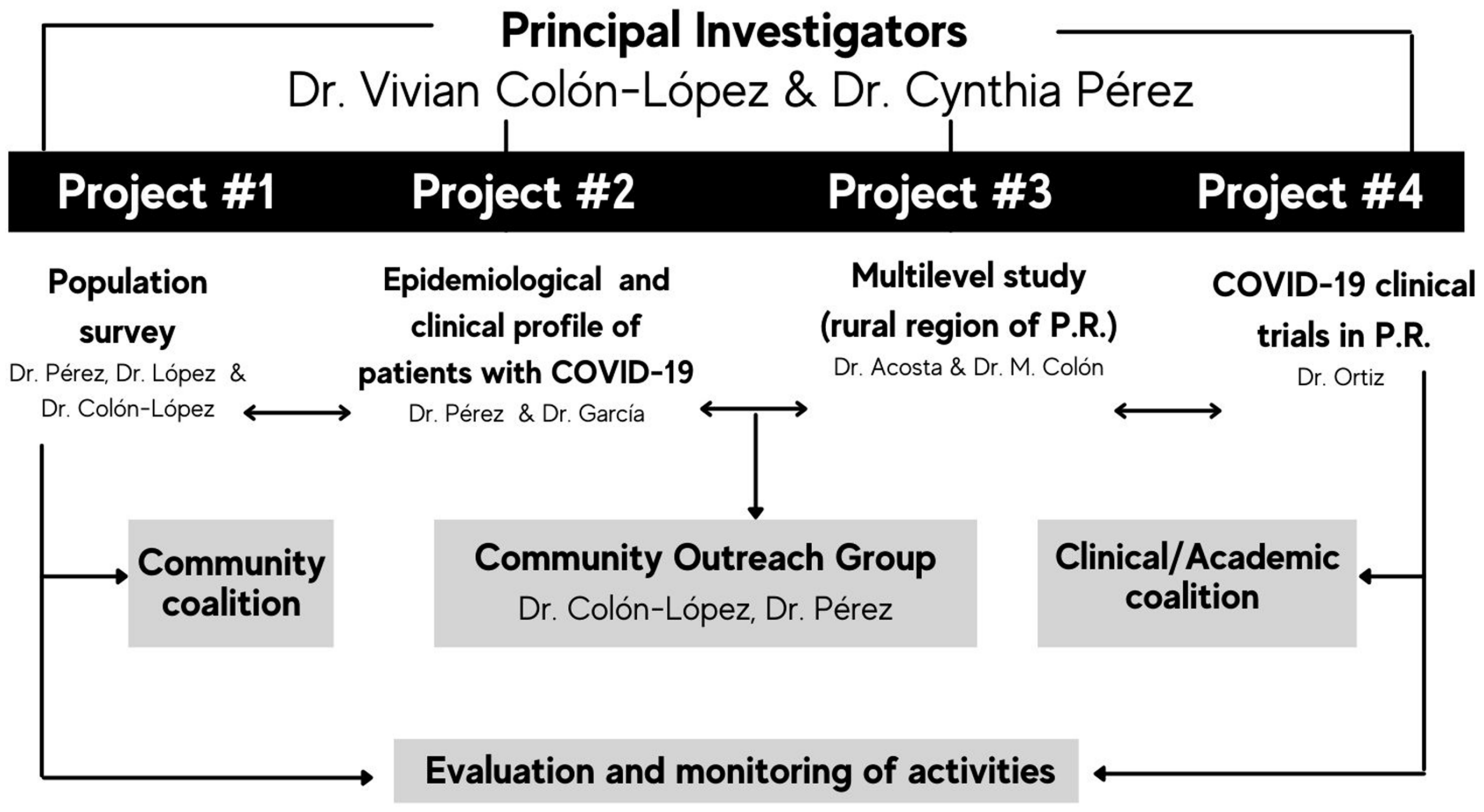
PR-CEAL infrastructure for Cycle 1.

During the inaugural year of funding, our primary objective at PR-CEAL was to spearhead urgent research initiatives to bolster mitigation efforts for COVID-19. This encompassed the development of prevention strategies and educational campaigns tailored to diminish distrust and misinformation surrounding this public health crisis. In its initial configuration, PR-CEAL comprised five main components: four standalone research projects and a Community Outreach Engagement Group (COEG). Additionally, PR-CEAL collaborated with two distinct, yet interconnected coalitions (Figure 1).

The four research projects and a community outreach component delved into critical areas for an immediate understanding of health disparities, emphasizing the dissemination and implementation of community-engagement strategies overseen by the COEG. The principal investigators who lead these efforts are leaders in the field with vast experience in infectious disease, epidemiology, Community-Based Participatory Research, and Community Outreach and Engagement. Support staff from PR-CEAL varies as more experienced staff were supporting the emergency efforts at the time. Research findings were deliberated with the two-coalitions. The clinical-academic coalition played a pivotal role in interpreting results and identifying impactful community strategies. Simultaneously, the community-advisory coalition, including stakeholders and community leaders, contextualized findings within their communities, considering emergent challenges.

**Figure 2:**
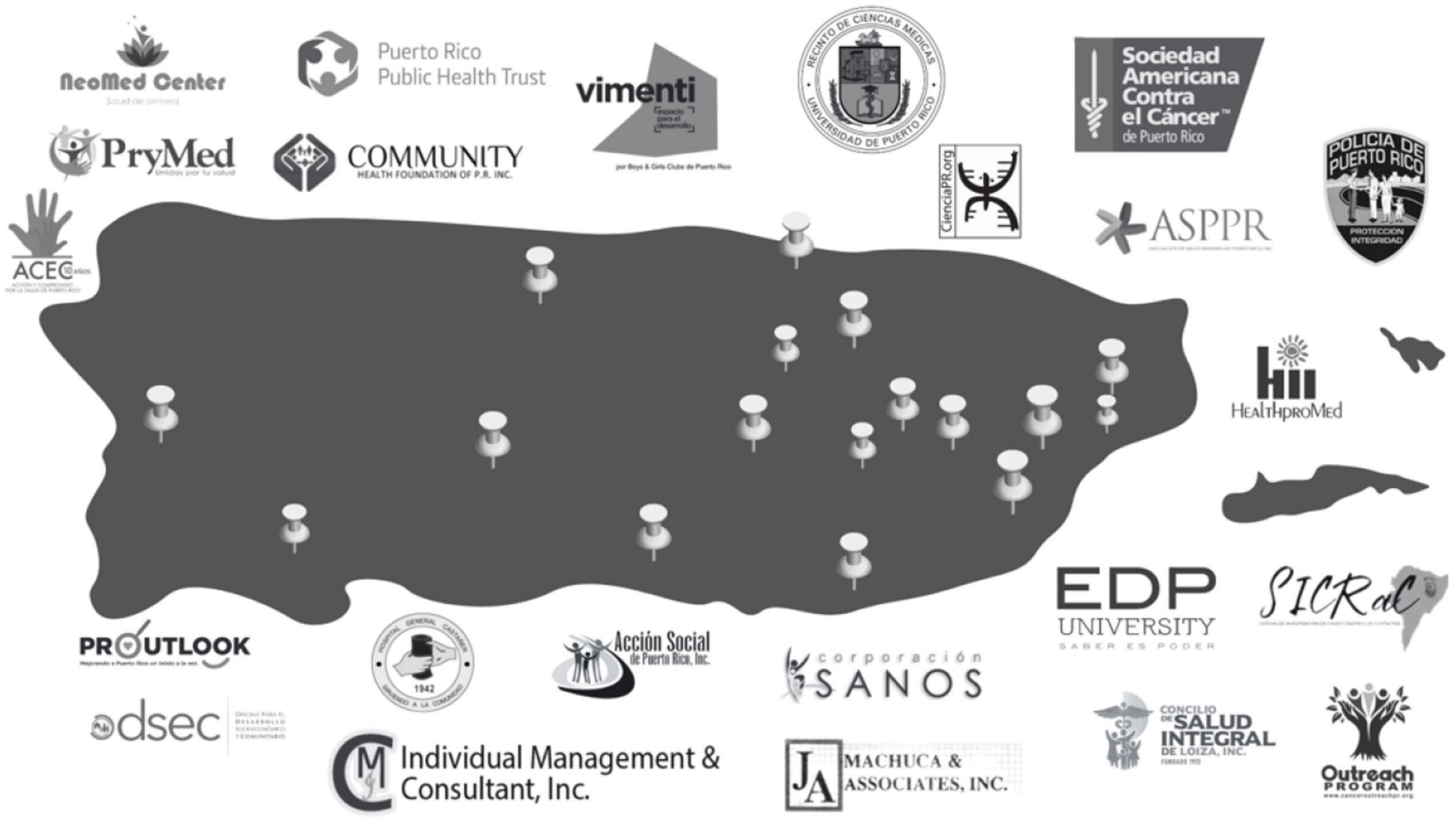
PR-CEAL: Community-Academic partnership.

Continuous research endeavors, coupled with the collaborative processing of data and addressing needs in tandem with the coalitions, paved the way for developing numerous successful community-based strategies islandwide. All these strategies were conceived in active collaboration with our community and clinical partners (Figure 2). The four research projects that were part of cycle 1 of PR-CEAL were the following:

#### 3.1.1 Project 1—Intention, beliefs, and predictors of vaccination against COVID-19 among adults in Puerto Rico

Project 1 was designed to comprehensively assess COVID-19 vaccine and booster uptake and attitudes and beliefs toward vaccination. The project also aimed to understand perceived barriers and enablers of vaccination, testing behaviors, and various modules related to health behaviors and their impact on mental health outcomes during the pandemic. Adopting a cross-sectional design, the study recruited 784 participants in Puerto Rico through online and in-person channels between December 2021 and January 2022 (9).

Collaborating with our nationwide CEAL partners, a concerted effort was made to develop a survey applicable across diverse settings. Employing the CEAL Common Survey, a cross-sectional study was conducted using a convenience sampling approach. The survey encompassed a range of variables, including sociodemographic characteristics, social determinants of health, COVID-19 prevention behaviors, testing behaviors, trusted sources of information about COVID-19, and general perceptions of the virus. The initial version of the survey was crafted in October 2021. Given the dynamic nature of the pandemic and the introduction of vaccines, a second version of the Common Survey was generated in December 2021. This revised edition incorporated questions about the intent to receive a COVID-19 booster vaccine, self-reported chronic conditions, influenza vaccine uptake, and cancer screening practices.

Building on the work of Wong et al. (21), PR-CEAL integrated additional questions based on the Health Belief Model (HBM) (22) to explore COVID-19 vaccination beliefs, attitudes, and behaviors. An analysis of the HBM constructs revealed that 83% of these constructs exhibited significant associations with booster refusal. Notably, participants who disagreed with the notion that receiving the booster dose made them feel less worried about COVID-19 or believed that the vaccine decreased their chances of contracting COVID-19 demonstrated higher prevalence ratios for booster refusal (9). Participants who expressed concerns about booster side effects, efficacy, and safety were significantly more likely to refuse the booster. Beyond vaccine-related factors, the dataset enabled analyses to explore challenges related to accessing basic needs such as healthcare services, medications, food, and water during the later stages of the pandemic (23). Furthermore, Project 1 uncovered a significant association between individual and external stressors and their impact on self-reported health. This study also revealed that a considerable proportion of participants, particularly women, experienced changes in stress (78%), weight (56.4%), and eating frequency (45.2%) during the pandemic. These findings underscore the multifaceted nature of factors influencing booster vaccine acceptance and refusal, as well as the broader impact of the pandemic on various aspects of individuals’ lives, health, and well-being.

#### 3.1.2 Project 2: Identifying communities that have been disproportionately affected during the COVID-19 pandemic

Project 2 aimed to enhance our understanding of the sociodemographic and clinical characteristics of suspected and confirmed COVID-19 patients receiving care at primary care clinics in Puerto Rico (24). Collaborating with four Federally Qualified Health Care Centers (FQHC) situated in low-income communities, this project sought to shed light on how the pandemic impacted vulnerable populations. The recruitment of suspected and confirmed COVID-19 patients occurred between April 1, 2020, and March 31, 2021. PR-CEAL staff visited the centers and performed medical record reviews to collect epidemiological and clinical information, resulting in a comprehensive database comprising 2,501 patients. Over half (53.8%) of the patients visiting the centers for COVID-19 testing were confirmed to have SARS-CoV-2 infection during the first year of the pandemic. Notably, compared to unconfirmed cases, those with confirmed infections were significantly younger (35.7 ± 19.1 years versus 42.7 ± 19.2 years). Confirmed cases also reported experiencing various symptoms, including cough, headache, muscle aches, and fever. The data also revealed a high prevalence of comorbidities among confirmed COVID-19 cases, with almost 50% of the patients reporting at least one chronic condition, including hypertension, mental health conditions, and diabetes. This insight into the sociodemographic and clinical characteristics of COVID-19 patients in primary care settings contributes valuable information for tailoring healthcare strategies and interventions to better address the needs of vulnerable communities during the ongoing pandemic.

#### 3.1.3 Project 3: Understanding community preparedness, barriers, and facilitators related to COVID-19: community participatory research in Castañer, Puerto Rico

Castañer, located in the central rural expanse of Puerto Rico, is recognized as one of the most geographically secluded communities in the island. It features its own hospital and benefits from a Health Resources and Services Administration (HRSA)-funded FQHC (25). Project 3 utilized a participatory comprehensive community-engaged, mixed-method, multilevel approach to examine barriers and facilitators in the patient, provider, and stakeholder spheres of the Castañer region (26). The methodology involved: (1) Qualitative Exploration: Three focus groups and 23 in-depth interviews, for a total of 56 participants, were conducted via face to face, virtual platforms, or telephone, engaging patients, providers, and key stakeholders within Castañer General Hospital (CGH) and its affiliated primary care clinics in Adjuntas and Jayuya. (2) Quantitative Survey Instrument: One hundred household-based quantitative surveys were systematically administered across four municipalities in the service region of CGH, namely Maricao, Jayuya, Adjuntas, and Lares. Survey participants were recruited through strategic locations like town squares, engagement with school personnel, and involvement with the extended family members of students.

The narratives from participants in the focus groups vividly highlighted the widespread impact of misinformation and mistrust surrounding COVID-19 vaccines (26). These sentiments, which centered on uncertainties regarding vaccine manufacturing, intended purpose, and overall credibility, emerged as significant barriers to broad vaccination efforts. One participant aptly expressed, *“[People would tell me] they are using us as guinea pigs, that [the vaccine] is still not approved. That is the type of misinformation many people received that prevented them from getting vaccinated. So then, we would get many people infected again. People would ask me: “how dare you get vaccinated?” And I would answer: ‘Well, because it is a resource available for everyone, so ‘let us use it’, but people still did not want to get vaccinated’.”*

Participants highlighted their primary concerns about family, community, and workplace. They discussed the need for continuous services for older adults lacking transportation, the challenge posed by low technological literacy, and the effects of the pandemic on children. These led to notable shifts in mental health and well-being due to the physical and emotional toll of the pandemic. *“[...] many patients express their depression is directly linked to the isolation imposed by the pandemic,”* a health personnel shared. They often say, “*I feel depressed because I cannot go out or my kids will not spend time with me*. *It feels like I cannot do anything; I am locked up all the time.”*

Most of the survey participants consistently followed the best COVID-19 public health practices, with 91% reporting steadfast mask usage, 84% practicing diligent handwashing, 72% maintaining physical distancing, and 68% actively avoiding crowded spaces. Notably, one in five participants tested positive for COVID-19 and among those who contracted the virus, one in 10 reported lingering effects. The vast majority (90%) found COVID-19 testing either ‘very easy’ or ‘easy’. Responses revealed a nuanced perspective regarding the perceived safety of available COVID-19 vaccines. Approximately 44% expressed confidence (felt somewhat safe), albeit tentative, while 26% conveyed a strong sense of assurance (felt very safe).

The findings underscored the necessity of combating misinformation, dispelling misconceptions, and mitigating message fatigue. This can be achieved by collaborative efforts with faith-based sectors, enhancing the skills of healthcare personnel, rather than solely focusing on emergency departments and employing individual and social behavior change models in crafting and delivering messages related to the protection, prevention, and management of COVID-19.

#### 3.1.4 Project 4: Promoting and facilitating the inclusion and diversity of Hispanics in clinical trials in Puerto Rico

Given that the COVID-19 pandemic spurred a surge in the initiation of treatment and vaccine clinical trials, this project compiled a directory of COVID-19 clinical trials in Puerto Rico and elucidated the perceived perspectives on participant recruitment among healthcare workers involved in conducting these trials on the island (27). The search from September 2021 to December 2021 utilized NIH RePORTER, clinicaltrials.gov, and centerwatch.com. Additionally, 14 professionals engaged in COVID-19 clinical trials were interviewed regarding details about the trials involved and queries about barriers and facilitators experienced in recruiting participants for these studies. A total of 23 COVID-19-related trials were identified, predominantly focused on treatment (73.9%) followed by vaccine development and efficacy (13.1%). Most trials were funded by pharmaceutical (34.8%) and biotechnology companies (30.4%), with 21.7% financed from federal sources (NIH and the Veterans Affairs Office). According to the interviewed research staff, participants agreed to join COVID-19 trials primarily if they had chronic diseases (92.9%) and because of the importance of clinical trials for developing an effective treatment for COVID-19 (85.7%) (27). Barriers to participation, as perceived by the research staff, included fear of side effects (85.7%), concerns about being treated as “guinea pigs” (71.4%), lack of interest (71.4%), time constraints (71.4%), among others (27). This data underscored the importance of enhancing efforts to promote and increase participant recruitment for COVID-19 trials in Puerto Rico.

This project also sought to understand why people participate in clinical trials using data from a cross-sectional study that collected information from 200 individuals aged ≥21 between November 2021 and March 2022 (28). Nearly all participants were vaccinated against COVID-19 (97.5%), and 65.5% had received the booster dose. Awareness of the availability of COVID-19 clinical trials was noted in only 63.5% of participants. In terms of willingness, 72.2% expressed that they would participate in a trial to receive COVID-19 treatment if infected (28). However, only 7.5% (*n* = 15) had been invited to participate, and merely 3.5% (*n* = 7) had participated in the trial. Among those who participated, common reasons included recognizing the importance of developing COVID-19 treatments and the desire to return to normalcy. Reasons for refusal to participate in a trial included a lack of sufficient information about the trial and concerns about treatment safety, among others (28). The study findings indicate interest in participating in COVID-19-related clinical trials among the studied population. Nevertheless, there is a need for increased efforts to raise awareness to enhance the likelihood of participation, particularly among Hispanic populations.

Given that cancer patients face heightened risks from severe COVID-19 complications, the project also examined COVID-19 clinical trial engagement, willingness, and trust among individuals with and without a history of cancer (29). Merging data from two cross-sectional studies conducted from November 2021 to March 2022 (*n* = 987), the study employed various interview methods with participants aged ≥18. Only 1.5% of the population participated in a COVID-19 clinical trial, with none of the cancer patients involved, and only 1.6% of non-cancer patients participated. While 37.0% expressed strong willingness to enroll in a COVID-19 treatment trial, willingness was higher in cancer patients (55.8%) than in those without cancer (36.1%) (29). Trust in information sources for clinical trials from cancer patients varied, with the highest levels observed at the NIH (90.2%), researchers (88.1%), physicians (85.4%), local clinics (80.9%), and a university hospital (78.6%) but lower for a pharmaceutical company (64.3%), and friend, relative, or community leader (40.5%); no differences were observed by cancer status (29). In conclusion, COVID-19 clinical trial participation was notably low, highlighting the need for education and awareness efforts, mainly targeting cancer patients to enhance representation among Hispanic and vulnerable populations for improved generalizability in future studies.

Finally, the public response to the COVID-19 pandemic has underscored the importance of trust, particularly among minority populations. Using data from PR-CEAL, we assessed the cross-sectional association between trust in information sources and COVID-19 vaccine trust in a sample of 200 adults living in Puerto Rico (30). Participants’ confidence in the safety of the COVID-19 vaccine was evaluated based on their level of certainty (“Not at all sure/Not very sure” or “Somewhat sure/Very sure”). Trust in COVID-19 information sources was assessed by the participants’ trust levels in that the information sources will provide accurate information of COVID-19, including the US-federal government, PR government, CDC, medical providers, and news (TV/radio/newspaper/internet; “Not at all/A little/Do not know” or “Very much”) (30). While most of the study sample (97.5%) had been inoculated with at least one dose of the COVID-19 vaccine, only 86% trusted the COVID-19 vaccine. After adjusting for age and sex, participants who attested greater trust in their medical providers (OR = 4.76, 95%CI: 1.95–11.61), the US federal government (OR = 6.62, 95%CI: 2.18–20.11), and the CDC (OR = 13.22, 95%CI: 5.21–33.53) reported increased vaccine trust as compared to those not having great confidence in these entities (30). These findings support that trust in information from US government agencies and medical providers is positively associated with COVID-19 vaccine trust. Acknowledging predictors of trust supports developing updated and new public health strategies to build vaccine confidence and benefit common welfare.

#### 3.1.5 Community Outreach Engagement Group (COEG)

**Figure 3:**
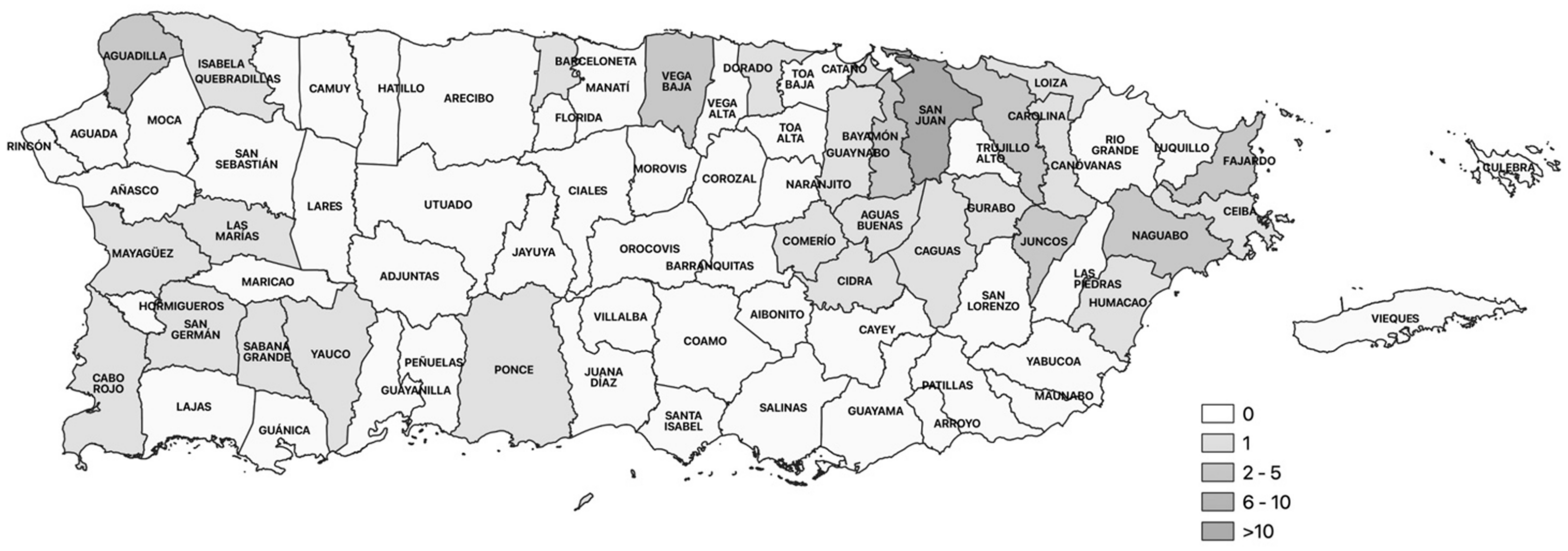
Municipalities reached by the community outreach engagement group.

The Community Outreach Engagement Group (COEG) is an integral component of PR-CEAL, focusing on translating research-driven information into accessible and actionable insights for communities. In pursuit of this objective, COEG has implemented various health promotion and education strategies across Puerto Rico aligned with the above mentioned projects and coalitions. During cycle 1, the COEG actively participated in 149 community events, demonstrating a dedicated commitment to community-engagement (31). Throughout this cycle, COEG focused on one-on-one educational support to counteract COVID-19 vaccine misinformation, debunking myths and emphasizing preventive practices such as disinfecting areas, physical distancing, handwashing, and mask usage. Moreover, COEG distributed resources like hand sanitizers and face masks in 54% of the municipalities in Puerto Rico, as illustrated in Figure 3. These community-events served as valuable platforms for gathering perspectives and concerns from the community, fostering a two-way communication. COEG utilized these interactions to share trusted, evidence-based resources, address the community’s needs, and dispel misinformation.

As part of this community-based effort, COEG also developed a survey tool to capture sociodemographic data from participants directly impacted by our educational activities. The first version of this survey, implemented in November 2021, focused on initial dose status, vaccine intentions, preferred vaccine manufacturer, booster eligibility, and influenza vaccine status and intentions. After new recommendations regarding boosters, a second survey version was adapted in December 2021. This revised version included questions about the intention to receive a booster and chronic diseases (i.e., diabetes, cancer, and liver disease) that could elevate the risk of severe illness if infected with COVID-19. The outreach team successfully engaged with 443 participants during this cycle using this survey-tool. The findings from this study played a pivotal role in shaping targeted communication strategies across communities, with a specific focus on enhancing education, prevention, and inclusion (31).

In addition to measuring beliefs, perceptions, and knowledge regarding vaccine uptake, the COEG developed a series of tailored educational programs targeted to the general population. In December 2021, the COEG launched its inaugural educational campaign, ‘Tú Eres Esencial’ (You Are Essential), to counter misinformation and curb the spread of COVID-19 (32). This initiative, developed in collaboration with reputable scientists and physicians, significantly contributed to evidence-based vaccination campaigns in Spanish. The campaign comprised 34 short educational-videos featuring Puerto Rican health professionals, including medical doctors, psychologists, medical students, and public health experts. Addressing common questions and dispelled prevalent myths about COVID-19 and the vaccine, ultimately aiming to reduce the spread of the virus. The campaign created educational resources for dissemination across Puerto Rico, collaborating with clinical, academic, and community partners. The campaign targeted older adults, individuals with chronic illnesses, immunocompromised patients, pregnant women, children, and adolescents. Resulting in the establishment of a digital library building a social media presence. The videos were uploaded to the PR-CEAL YouTube page and shared across social media platforms, reaching nearly 3,000 individuals. The campaign played a positive role in increasing vaccine uptake, encouraging communities to stay current with COVID-19 vaccinations, receive booster doses, undergo COVID-19 screening tests, and understand and prevent prolonged COVID-19 conditions.

### 3.2 PR-CEAL Cycle 2 (April 2022–July 2023)

Building upon the evolving landscape of the pandemic and drawing insights from the data collected through research projects and community engagement, PR-CEAL outlined new objectives for Cycle 2. The aims encompassed the development of a novel component focused on health promoters and the collaborative support of other Community-Based Organizations (CBOs) across the island. The overarching goal was to expand the scope of strategies and engage with diverse subgroups while maintaining a central theme. The specific aims for Cycle 2 included:

1. Conduct community-engaged research and outreach activities focused on COVID-19 awareness, testing, vaccine booster, long COVID-19, and new variant infections.
2. Support community outreach initiatives in vulnerable and underserved populations by creating a pilot program to enhance the scope and reach.
3. Support ongoing NIH-funded research projects in PR aimed at improving recruitment strategies.
4. Conduct clinic-based research to understand the epidemiology, access to care, and quality of life of patients with COVID-19.

As of July 2022, Puerto Rico has achieved one of the highest COVID-19 vaccine uptake rates. However, despite this success, only 53.1% of the population had received booster doses (20), a particularly concerning statistic given the high burden of chronic diseases. In response, PR-CEAL, in collaboration with its community coalition and a communication agency, initiated a second educational campaign called ‘Con Refuerzo’ (Boosted) (33). The campaign aimed to complement Puerto Rico’s public health efforts by issuing a call to action for individuals who had received their primary vaccine doses to get booster shots. Additionally, the campaign encouraged those who received boosters to inspire others, including family, friends, and acquaintances, to follow suit. ‘Con Refuerzo’ utilized various mediums, including billboards, social networks, videos, and radio spots, to emphasize the critical importance of receiving booster doses. The campaign featured P.J. Sin Suela, a renowned exponent of urban music who also practices medicine in Puerto Rico, as its spokesperson. Leveraging P.J. Sin Suela’s dual role as a prominent musician and medical professional provided a unique opportunity to connect with and motivate the Puerto Rican population. Notably, this collaboration aimed to encourage individuals to act by getting their booster shots, focusing on reaching as many people as possible before P.J. Sin Suela’s August 2022 concert.

Continuing our commitment to health promotion and education, the COEG team initiated the ′Retomando mi Salud′ (Regaining My Health) campaign, officially launched in February 2023 (34). This campaign leveraged our social media platforms and a dedicated supplement in a local newspaper to disseminate its message. The primary goal of this campaign was to inspire individuals to prioritize their health by attending scheduled medical appointments and seeking screenings, prevention measures, diagnoses, and treatment for chronic illnesses.

To expand the impact of our community outreach efforts, PR-CEAL initiated a pilot program focused on supporting the development of community initiatives. Three non-profit community-based organizations, namely *Coalición de Coaliciones Pro Personas Sin Hogar*, *CienciaPR*, and *Arecma Inc.*, were recipients of grants from PR-CEAL. These grants were allocated to facilitate the creation of community initiatives specifically designed to address COVID-19 prevention and misinformation within Puerto Rico’s vulnerable and underserved populations. The selected organizations pursued distinct objectives aligned with their unique strengths and target communities, including homeless populations in Puerto Rico, reaching a diverse community through science, and impacting the rural region in the southeast of the island.

In response to the lack of comprehensive information on Long COVID within underserved populations in Puerto Rico, we conducted a prospective cohort study in 2022 focusing on individuals with confirmed SARS-CoV-2 infection (35, 36). Initial interviews covered sociodemographic details, comorbidities, vaccination status, and persistent or new symptoms post-acute infection. Out of 263 participants, 256 had the infection at least 4 weeks before the interview. The mean age of the participants was 48.1 ± 15.9 years, 201 (78.8%) were women, and 191 (74.6%) were presenting Long COVID symptoms. Those with Long COVID experienced a significantly higher prevalence of tiredness, muscle pain, headaches, joint pain, and anxiety. Adjusting for age, sex, and severity, having a chronic condition was significantly associated with higher odds of Long COVID (OR 3.38; 95% CI, 1.62–7.04). Specifically, individuals with asthma, obesity, hypertension, and diabetes had significantly higher odds of Long COVID. Preliminary data reveals a notable prevalence of Long COVID, particularly among those with comorbidities in Puerto Rico, indicating an elevated risk in this population.

**Figure 4:**
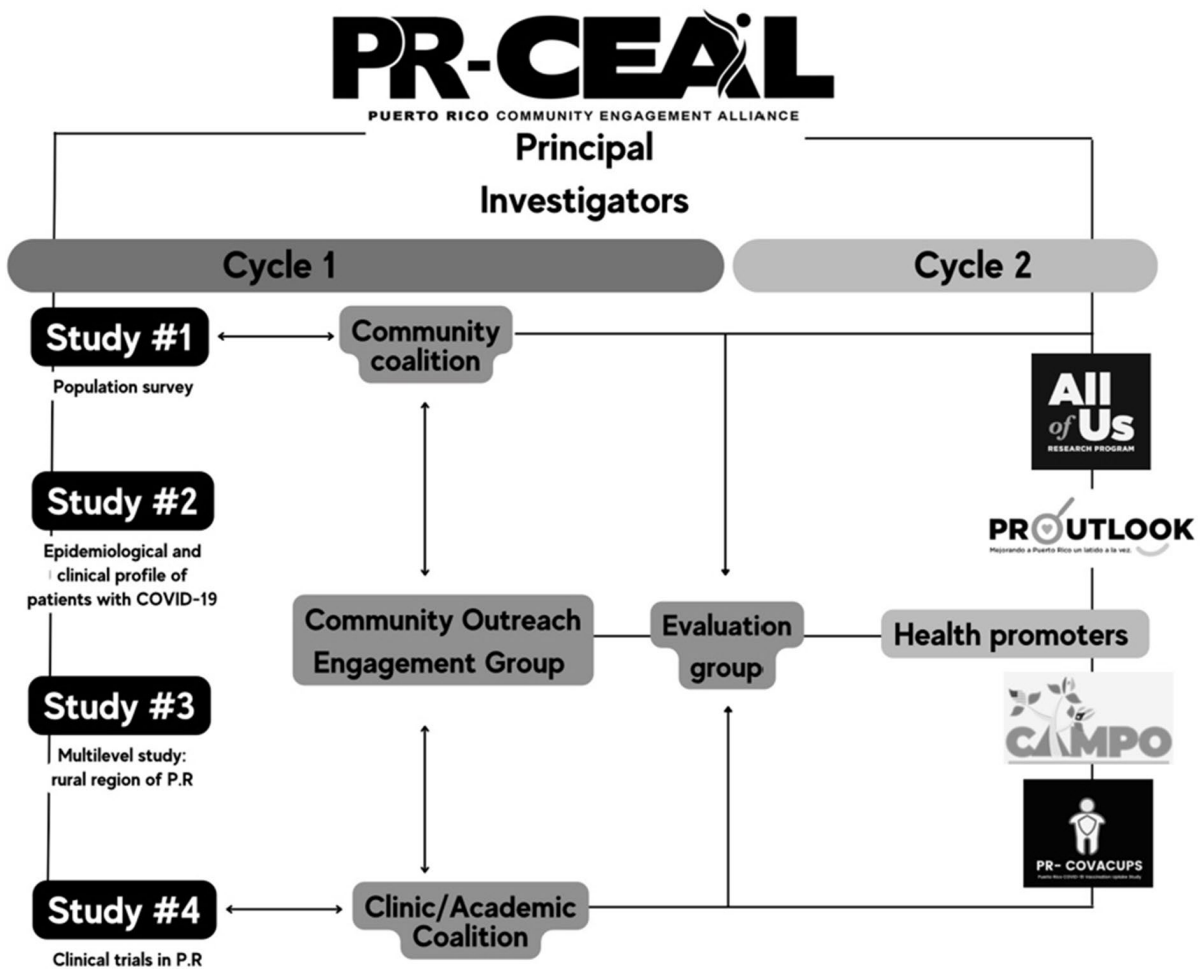
PR-CEAL infrastructure for Cycles 1 and 2.

A new health promoters’ component was developed to address the impact of the COVID-19 pandemic on NIH-funded research studies and to enhance the participation of Puerto Ricans in clinical trials and other initiatives. Recognizing the increased risk for severe disease and the underrepresentation of Puerto Ricans in research studies, the health promoters collaborated with the recruitment, in-person assessment, and follow-up of study participants in Puerto Rico, as illustrated in Figure 4. Several NIH-funded research studies benefitted from the support and involvement of the health promoters’ component, including PR-OUTLOOK (Puerto Rico Young Adults Stress, Contextual, Behavioral and Cardiometabolic Risk) (37), the California-Mexico-Puerto Rico (CAMPO) partnership center (38), All of Us Research Program (39), and Puerto Rico COVID-19 Vaccine Uptake Study (PR-COVACUPS) (40). The health promoters actively engaged with these research studies’ recruitment and retention support. Additionally, mutual support was evidence with the Community Engagement and Outreach Core of the Hispanic Alliance in Clinical and Translational Research (Clinical and Translational Research Network, Institutional Development Award). Through their efforts, many interested individuals, totaling 3,907, were reached in various community settings. The health promoters played a crucial role in informing individuals about opportunities for participation in clinical trials or population-based research, contributing to the broader goal of increasing representation and engagement in research studies among the Puerto Rican population. This collaborative approach ensures that research outcomes are more inclusive and representative of diverse communities.

## 4 Discussion

The CEAL initiative has successfully implemented an integrated infrastructure that enhances collaboration between scientists and community-based partners nationwide. This infrastructure enables the rapid development of evidence-based community engagement responses to address and reduce disparities in disease outcomes. Specifically, for PR-CEAL, the initiative has strengthened ties with community partners, promoted bilateral communication between academia and the community, and fostered a valuable partnership that allowed us to achieve our goals and pinpoint areas requiring attention. It has also expanded our network with new community partners and organizations that serve specific groups and communities, which has helped to increase the reach of vulnerable and underserved communities. These vulnerable, non-institutionalized populations include the older adults, homeless, children, immunocompromised individuals, families living in poorer areas, caretakers of functional diversity patients, and pregnant women. The COEG team reached these groups during various activities and project-related initiatives throughout the island. During our collaboration with Coalición de Coaliciones Pro Personas Sin Hogar, Inc., homeless individuals o in the South Region of Puerto Rico were impacted as the team provided COVID-19 prevention materials, clothing, and evidence-based information of COVID-19. However, several limitations posed challenges to establishing collaborations with certain organizations to address needs effectively. Specifically, constraints on the financing period, limited from the CBO and limited administrative support for the allocation of funds for team personnel contracts, creates gaps in an expedite collaboration with our partners. Despite limited funding due to the timeline of the grant, community-based relationships can be sustained with strategic planning and capacity building.

Moreover, through dynamic exchanges with our partners, we bridged the gap between theoretical insights and practical applications, ensuring that our initiatives had a meaningful impact on people living in Puerto Rico. Our community partners provided valuable insights, real-world perspectives, and a deeper understanding of the challenges faced by these communities. Meanwhile, the academic community contributed expertise, cutting-edge research, and extensive resources. Information gleaned from research projects and community interactions has been instrumental in understanding the challenges faced by different communities and establishing trust in the scientific process. Furthermore, these data have been crucial in targeting COVID-19 resources equitably to each community.

These initiatives have not only shed light on effective ways to address challenges but have also contributed to understand the burden of COVID-19 in underserved communities. Lessons learned led to a robust infrastructure to efficiently address a variety of public health inequities, from a local context perspective, in collaboration with our community partners. Insights have identified information-based needs and perceptions about vaccines and treatments, addressing concerns about medical mistrust and healthcare access inequities. These findings have been instrumental in guiding future community outreach initiatives and strategic action. The collected data serves as a valuable tool for decision-makers, enabling them to analyze and understand the community’s unique needs and challenges, and have served as a learning lesson to scientists in conveying community partners into research protocols since its inception.

It has also promoted a continued dissemination of findings, leading to new initiatives that are research-driven and value-added for investment in our community partners. With this infrastructure, future efforts from CEAL will implement evidence-based interventions to address Social Determinants of Health in underserved communities nationwide. PR-CEAL will focus on health literacy, food insecurity, and access to healthcare as we aim to help break down the systematic barriers that contribute to health disparities. Community outreach and engagement will be at the core of our approach, ensuring that interventions are culturally tailored to assess specific community needs.

## 5 Conceptual or methodological constraints

While PR-CEAL has made significant strides in its community engagement efforts, it is crucial to acknowledge the challenges and limitations encountered, which can inform and guide future projects that provide rapid responses. The spread of the Omicron variant and adherence to social distancing guidelines posed significant challenges to data collection settings and methods. Participant recruitment faced hurdles due to restrictions and safety concerns associated with the evolving pandemic situation. Additionally, Project 4 encountered recruitment challenges due to the resistance from community members stemming from a lack of trust in participating in research initiatives and clinical trials. However, different mechanisms were implemented to overcome these challenges, utilizing typical recruitment venues such as flyers, official university e-mail blasts, and crowdsourced research platforms. The dynamic nature of COVID-19 guidelines, including mask recommendations, vaccination protocols, social distancing measures, the spread of misinformation, and the uncertain and prolonged pandemic timeline, introduced challenges in planning and executing projects. Moreover, these continuous preventive guidelines caused pandemic fatigue among Puerto Ricans, disrupting health promotion practices for COVID-19. Furthermore, due to the limited availability of ferry transportation, we were unable to impact the municipalities of Vieques and Culebra, two nearby islands that are off the east coast of Puerto Rico. Future projects should consider these challenges and proactively plan for strategies to mitigate their impact. Flexibility in project design, robust communication and dissemination strategies, and ongoing support for community health workers are essential for success in the rapidly evolving landscape of public health emergencies.

## Data availability statement

The original contributions presented in the study are included in the article/supplementary material, further inquiries can be directed to the corresponding author.

## Ethics statement

The studies involving human participants were reviewed and approved by the University of Puerto Rico Institutional Review Board (Protocol # A806022). Written informed consent for participation was not required for this study in accordance with the national legislation and institutional requirements.

## Author contributions

AP-C: Writing – original draft, Conceptualization, Data curation, Formal analysis, Methodology, Writing – review & editing. CP: Writing – review & editing, Conceptualization, Data curation, Writing – original draft, Methodology. KC-B: Writing – review & editing, Data curation, Formal analysis, Methodology. ZT-Q: Writing – review & editing. NS-T: Writing – review & editing, Data curation, Formal analysis, Methodology. AL-C: Writing – review & editing, Conceptualization, Data curation, Formal analysis, Methodology. EG-R: Conceptualization, Data curation, Formal analysis, Writing – review & editing, Methodology. ML: Writing – review & editing. EA-P: Conceptualization, Data curation, Formal analysis, Writing – review & editing, Methodology. MC: Conceptualization, Data curation, Formal analysis, Writing – review & editing, Methodology. ZR: Data curation, Formal analysis, Writing – review & editing. AO: Writing – review & editing, Conceptualization, Data curation, Formal analysis, Methodology. FR-G: Writing – review & editing. VC-L: Writing – review & editing, Conceptualization, Data curation, Writing – original draft, Methodology.
